# A Review of the History of Radioactive Iodine Theranostics: The Origin of Nuclear Ontology

**DOI:** 10.4274/mirt.galenos.2020.83703

**Published:** 2020-10-19

**Authors:** John Dennis Ehrhardt Jr, Seza Güleç

**Affiliations:** 1 Miami Cancer Research Center, Miami, USA; 2 Aventura Hospital and Medical Center, Miami, USA; 3 Florida International University Herbert Wertheim College of Medicine, Miami, USA

**Keywords:** Radioactive iodine, theranostics, nuclear oncology, Saul Hertz, Irving Ariel, Samuel Seidlin, Henry Beierwaltes, thyroid cancer, genomics

## Abstract

Studies on the first years of radioactive iodine (RAI) use in thyroid diseases have focused on hyperthyroidism. Saul Hertz’s success with RAI in thyrotoxicosis fueled a seamless transition to Samuel Seidlin’s investigations with RAI in thyroid cancer. These landmark events embody nuclear ontology, a philosophical foundation for the creation and existence of radio-therapeutic principles that continue to influence clinical practices today. Laying this ontological foundation, Dr. Saul Hertz who is the founding director of Massachusetts General Hospital Thyroid Clinic, affiliated with Harvard University created a framework for RAI theranostics with preclinical experiments and clinical cases from 1937 to 1942. The first thyroid cancer treatment with RAI was applied in 1942 by Samuel Seidlin. The sensational effect of the first application was interestingly powerful enough to overshadow scientific data. Seidlin and colleagues assembled a sixteen-patient series showcasing a unique entity: functional thyroid metastases that respond to RAI. Other investigations at the time demonstrated that RAI had little efficacy as a therapeutic agent, mainly because most thyroid tumors do not form colloid, and therefore cannot concentrate RAI. These findings were soon overshadowed by a mainstream article in the October 1949 issue of Life that portrayed RAI as a lifesaving therapy for thyroid cancer. The paradigm was set, and later writings by William H. Beierwaltes and other prominent nuclear medicine physicians established the primary goals and principles of RAI therapy. The developments in theoretical physics and nuclear instrumentation and the scientists who made these developments in the early years contributed greatly to the development of the concept. In the field of nuclear medicine, William H. Beierwaltes has gone down in our history as a clinical researcher with his most important contributions. The classical paradigm that started with him has carried us to today’s molecular theranoistic viewpoint. This paper examines controversial topics in the advent of thyroid theranostics, and applies historical significance to current discussions on the role of RAI in thyroid cancer management. Another paradigm shift is on the horizon as thyroidology enters the age of genomics. The molecular theranostic profiles will soon be incorporated into a dynamic clinical decision-making and management algorithm for thyroid surgery and RAI therapy. From now on, nuclear oncology will gain a new ontological identity with molecular pathology and new theranostic expansions.

## Abstract (tr)

Tiroit hastalıklarında radyoaktif iyot (RAI) kullanımının ilk yıllarına ait çalışmalar hipertiroidi konusunda yoğunlaşmıştır. Saul Hertz’in tirotoksikozda RAI ile elde ettiği başarı, Samuel Seidlin’in tiroid kanseri tedavisi üzerinde RAI araştırmalarını kolaylıkla tetiklemiştir. Bu dönüm noktası niteliğindeki olaylar, günümüz klinik uygulamalarını etkileyen radyoterapötik ilkelerin oluşturulması için felsefi bir temel olan nükleer ontolojiyi somutlaştırmıştır. Bu ontolojik temeli atan Dr. Saul Hertz, Harvard Üniversitesi ile bağlantılı çalışan Massachusetts General Hospital Tiroit Kliniği’nin kurucu direktörüdür. Hertz preklinik deneyler ve klinik olgular ile, bugünkü bilgilerimize esas teşkil eden tiroid teranostik çalışmalarını 1937-1942 yılları arasında gerçekleştirmiştir. RAI ile tiroit kanseri tedavisi ilk kez 1942 yılında Samuel Seidlin tarafından uygulanmıştır. İlk uygulamanın sansasyonel etkisi, ilginç olarak, bilimsel verileri gölgeleyecek denli güçlü olmuştur. Seidlin ve meslektaşları, benzersiz bir durumu sergileyen on altı hasta serisini bir araya getirmiştir: RAI’ye yanıt veren fonksiyonel tiroit metastazları. O zamanki diğer araştırmalar, RAI’nın terapötik bir ajan olarak etkinliğinin az olduğunu göstermiştir, çünkü çoğu tiroit tümörleri kolloid oluşturmaz ve bu nedenle RAI’yı konsantre edemezler. Bu bulgular kısa süre sonra Life dergisinin Ekim 1949 sayısında RAI’yi tiroid kanseri için hayat kurtaran bir tedavi olarak tasvir eden ana akım bir makale tarafından gölgede bırakılmıştır. Ardından paradigma belirlenmiş ve daha sonra William H. Beierwaltes ve diğer önde gelen nükleer tıp doktorlarının yazıları ile RAI tedavisinin birincil hedef ve ilkeleri belirlenmiştir. Kavramın gelişmesinde, erken yıllarda teorik fizik ve nükleer instrumentasyonlardaki gelişmelerin ve bu gelişmelere imza atan bilim adamlarının büyük katkıları olmuştur. Nükleer tıp alanında ise en önemli katkılarıyla William H. Beierwaltes bir klinik araştırmacı olarak tıp tarihine geçmiştir. Onunla başlayan klasik paradigma, bizleri bugünün moleküler teranostik bakış açısına kadar taşımıştır. Bu makale, tiroid teranostiklerinin ortaya çıkışındaki tartışmalı konuları incelemekte ve RAI’nın tiroid kanseri yönetimindeki rolü konusundaki güncel tartışmalarda tarihsel önemi vurgulamaktadır. Tiroidoloji genomik çağına girerken bir başka paradigma dönüşümü ufuktadır. Moleküler teranostik profiller yakında tiroid cerrahisi ve RAI tedavisi için dinamik bir klinik karar verme ve yönetim algoritmasına dahil edilecektir. Bundan böyle moleküler patoloji ve yeni teranostik açılımlar ile, nükleer onkoloji artık yeni bir ontolojik kimliğe kavuşacaktır.

Today is only one day in all the days that will ever be. But what will happen in all the other days that ever come can depend on what you do today”.

**- Ernest Hemingway**

## Radio-iodine Halts One Type of Cancer

**Figure 1:**
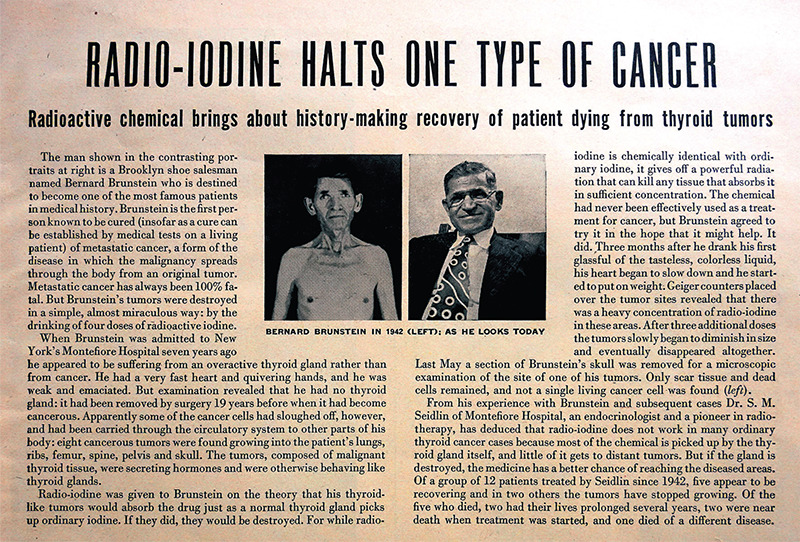
A popular article featuring Samuel Seidlin’s famous patient who was “cured” of thyroid cancer by radioactive iodine therapy, dated October 31, 1949 *(Life Magazine)*

On October 31, 1949, the *Life *Magazine ran an article titled “Radio-iodine Halts One Type of Cancer” with the subheading “Radioactive chemical brings about history-making recovery of patient dying from thyroid tumors” (1). This article (Figure 1) featured the “before and after” photographs of Bernard Brunstein, the Brooklyn shoe salesman who was “cured” of thyroid cancer with the use of radioactive iodine (RAI). The “before” photograph from the year 1942 shows him as cachectic and frail, which is in stark contrast with the “after” photograph, which showcases him as a healthy-appearing man with a full face. This convincing portrayal of RAI as a miraculous therapy for thyroid cancer left a strong impression on the public and practicing clinicians. The article’s profound rhetoric permeated medical teaching and influenced clinical reasoning, which soon placed RAI treatment as the core modality for the management of “differentiated” thyroid cancer. RAI, which is the first theranostic radiopharmaceutical approach, has ontological origins that date back to November 12, 1936, which forever changed the management paradigm for thyroid diseases.

## Saul Hertz, Father of Theranostics

**Figure 2:**
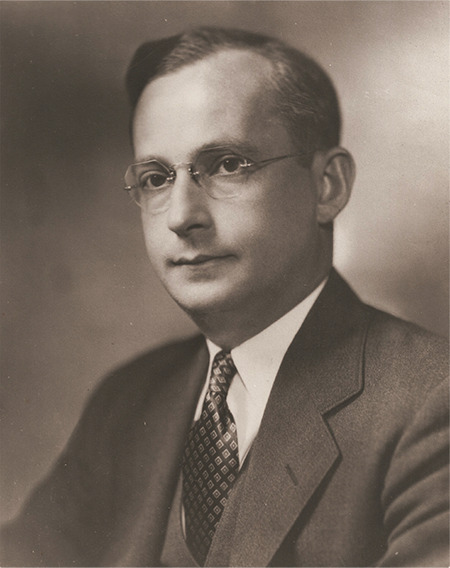
Saul Hertz, Chief of the Thyroid Clinic at the Massachusetts General Hospital (MGH) who introduced the concept of RAI theranostics

**Figure 3:**
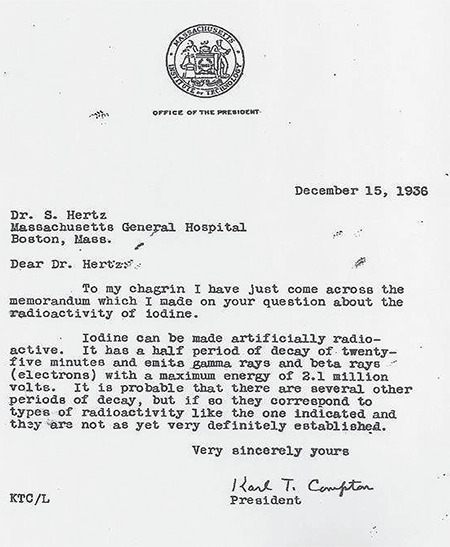
Massachusetts Institute of Technology (MIT) president Karl Compton’s letter to Saul Hertz, the undisputable proof that Hertz is the father of Nuclear Theranostics

Saul Hertz (Figure 2), Chief of the Thyroid Clinic at the Massachusetts General Hospital (MGH), joined the Chief of Medicine, James H. Means, for a luncheon at the Harvard Medical School’s Vanderbilt Hall on November 12, 1936. The keynote speaker was Massachusetts Institute of Technology (MIT)’s President Karl Compton, whose talk on “What Physics Could do for Biology and Medicine” sparked Hertz’s creativity. From the audience, Hertz asked, “Can iodine be made artificially radioactive?” He was already several years deep into experiments on thyroid iodine physiology at the MGH in his quest for a non-operative solution to hyperthyroidism. Compton was unsure how to answer, maybe partially because his lecture’s focus on artificial radioisotopes came recommended last-minute by the MIT physicist Robley Dunglison Evans. Nonetheless, Compton jotted a reminder to himself and wrote to Hertz a month later on December 15, 1936 (Figure 3): “To my chagrin I have just come across the memorandum which I made on your question about the radioactivity of iodine. Iodine can be made artificially radioactive. It has a half-period of decay of twenty-five minutes and emits gamma rays and beta rays (electrons) with a maximum energy of 2.1 million volts. It is probable that there are several other periods of decay, but if so they correspond to types of radioactivity like the one indicated, and they are not as yet very definitely established” (2).

To this, Hertz replied 8 days later on December 23, 1936, hinting at his hypothesis, he wrote “…hope that iodine, which is made radioactive as you indicated will be a useful method of therapy in cases of over-activity of the thyroid gland”. From a historical perspective, the correspondence between Compton and Hertz is an indisputable proof that the idea of RAI theranostic arose from Hertz. The Hertz family later released these letters; however, several histories, even 21^st^ century publications, have presented the 1936 Vanderbilt Hall interaction in a way that credited the Chief of Medicine J.H. Means for presenting the RAI idea (3,4,5). We can thus be sure, beyond any reasonable doubt, that it was Saul Hertz who posed the question first.

Hertz’s RAI research work began in the early 1937. Then, Evans was appointed to manage the physics laboratory at MIT, where we designed for the small-scale production of non-cyclotron ^128^I. The experimental protocol followed was the one proposed by the 1934 *Nature* paper article by Fermi (6), an Italian-American physicist who later became famous as the architect of the atomic bomb. Evans recruited a junior faculty, the MIT nuclear physicist Arthur Roberts to perform the grunt work of isolating ^128^I. Nearby at the MGH, James H. Means, who had previously told Hertz that he intended to let his contract expire at the thyroid clinic, renewed Hertz’s directorial position and charged him with the job of overseeing all biological and medical aspects of the radioiodine project. Under this Hertz-Roberts collaboration, an experimental study of 48 rabbits was first undertaken, which demonstrated that the goitrous thyroid glands retained more RAI than the normal control glands. They ran experiments at the MIT because the 25-minute half-life of ^128^I prohibited transportation of the radionuclide across the Charles River to the MGH. The scholars later speculated that their original experiment produced only 0.05 miliCurie (mCi) of activity, which is extremely small in comparison with the 100-mCi therapeutic activity that is commonly applied presently for RAI treatments (7). Although successful in laboratory, Hertz realized that the small experimental yield and the short half-life of ^128^I made its clinical application impractical. To ramp up the production, the MIT outlined plans to build a cyclotron for the mass creation of radioiodine. The then President Compton and the Hertz-Roberts laboratory manager Evans took a day trip to Manhattan in May 1938 and secured a $30.000 grant (equivalent of $530.000 today) from the John and Mary Markle Foundation to fund the proposed MIT cyclotron. Construction was arduous and MIT soon consulted the University of California at Berkeley, where Ernest O. Lawrence’s original cyclotron possessed novel capabilities to create different radioisotopes, each with longer half-lives than ^128^I. Building the MIT cyclotron was believed to solve the problem of experimental yield, albeit solutions for short half-lives remained unresolved.

Lawrence’s cyclotron had established the University of California at Berkeley as the west coast hub for radionuclide research. The work progress continued when Glenn Seaborg and Jack Livingood discovered the popular ^131^I there in 1938 by irradiating tellurium targets (8).  This breakthrough came after a conversation between Seaborg with fellow laboratory associate Joseph A. Hamilton, a physician-scientist who explained that the short half-life of ^128^I placed great constraints on its clinical application. In collaboration with Livingood, Seaborg then produced ^131^I roughly 1 week after that his dialogue with Hamilton. Several of the radioisotopes discovered at the Berkeley cyclotron, including ^131^I, were pursued out of passion for pure physics. Seaborg later reflected that they “usually gave little thought to the possibility that one of the objects of our search would have practical value. But we were in for some surprises” (9). However, the l arge-scale synthesis of ^131^I remained a problem until 1941. Seaborg could live to see the impact of his contribution to thyroid diseases when his mother later required RAI treatment for hyperthyroidism (10). The fission-derived radioiodine was made freely available in the year 1946 as a consequence of the Manhattan project in the “secret town of Oak Ridge” in Tennessee.

**Figure 4:**
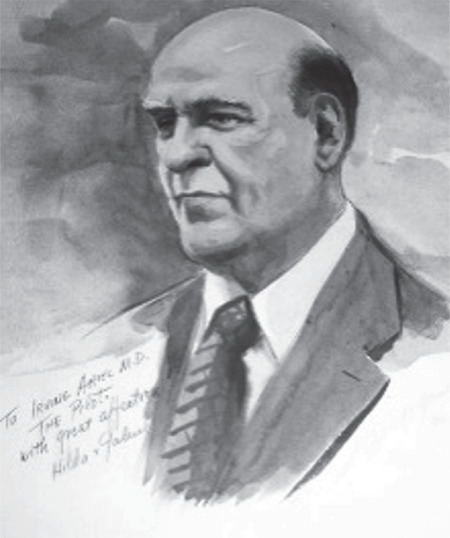
Irving Ariel, a New York Surgeon, who is well recognized for his clinical and translational Nuclear Medicine research introduced I-131 to the surgical world

Despite all the technical hitches, the RAI research continued to expand. Irving Ariel, a surgeon by occupation, performed animal studies using 12.5-hour half-life I-130 at the University of Rochester. Ariel (Figure 4) later continued his clinical and research studies in New York’s Memorial Hospital. Ariel’s career as a “Nuclear Surgeon” flourished in the later years, and he became best known as an innovator in the radiomicrosphere therapy (11). In fact, he was among the first to shift to ^131^I when it became more available. The Ariel group later demonstrated the clinical importance of dose rate, which suggested that ^131^I had a higher therapeutic potential than other radioisotopes with longer half-lives, namely the 60-day ^125^I (12). In addition, Ariel spent most of his career as a clinician and a professor of surgical oncology in New York, affiliated with the Memorial Hospital and the Albert Einstein College of Medicine. The surgeon was one of the founding members of the Society of Nuclear Medicine. He also served as the trustee of the National Society of Nuclear Medicine and the president of the Greater New York Chapter.

At this juncture, radioiodine from the original cyclotron, for which Lawrence later won the 1939 Nobel Prize in Physics, became available as ^126^I (t_1/2_=13 days) and ^131^I (t_1/2_=8 days), which were ready-to-ship first-class from Berkeley to Cambridge (13). This Berkeley-MIT-MGH collaboration continued for nearly 2 years until the MIT cyclotron opened in 1940. Distinct from other cyclotrons, MIT devoted theirs to biological and medical research, albeit for a profit. Evans initially charged researchers $25 for radiotracers from the newly minted MIT radioactivity center. He later admitted that this became their primary revenue source, generating over $88.000 by 1945, largely paid through federal military research contracts at the indirect expense of taxpayers. In contrast, Lawrence’s Nobel Prize-winning cyclotron at Berkeley continued shipping radioisotopes for free throughout the Second World War, supporting biomedical research as well as the Manhattan Project toward developing the atomic bomb (14).

Longer half-life ^131^I shipped to MIT throughout the years 1938-1939 allowed Hertz and Roberts to confirm radiotracer avidity in normal rabbit thyroid and goiter. However, one mishap occurred during these early experiments when the amount of stable-carrier iodide was administered with the radioiodide. The scholars never foresaw that stable iodide would compete for thyroid uptake and thereby reduce the radionuclide tissue penetrance. As a result, their dosage predictions for future clinical trials were exorbitantly high, estimated at 750 mCi for the effective treatment of hyperthyroidism (7).

The Hertz-Roberts team was academically productive, which was a blessing and a curse that strained relationships with the supervisors. For instance, their 1938 manuscript was intercepted after acceptance because they had neglected to add the lab supervisor of Evans as a co-author, despite the fact that he reportedly contributed nothing to the experiments, analysis, or scientific writing process. Evans forced Hertz to draft a letter to the editors requesting an amended author list (15). Their subsequent 1940 manuscript included both Evans and Means as co-authors, with Hertz and Roberts at the helm, independently operating their own laboratory. This glimpse of academic hierarchy foreshadowed what was yet to come. Scholarly disputes festered against the backdrop of rising political concerns-the rise of the Third Reich, palpable antisemitism, and radio broadcasts bringing battlefield scenes into American homes-all while two of the greatest thyroid investigators, Hertz and Roberts, were Jewish.

## The War and Subsequent *JAMA* Controversy

Hertz and Roberts began translating knowledge from their rabbit experiments to patients at the thyroid clinic. Hertz treated their first patient on March 31, 1941 and documented the process in his handwriting as “Elizabeth D.”, with 2.1-mCi RAI. Hertz’s case logs of RAI treatments grew steadily, with the addition of roughly 1 new patient every month. By 1943, his series had grown to include 27 patients with hyperthyroidism who were treated solely with RAI. His term as the director of the thyroid clinic ended during the summer of 1942. Means, however, agreed to pay Hertz a partial salary to continue his hyperthyroidism research. Thus, Hertz transitioned to private practice by opening his own Boston clinic from which he occasionally funneled patients into his ongoing RAI case series. At this time point, his MIT research partner Roberts was no longer involved, as he had turned his focus to other projects in particle physics and never returned to the thyroid subject again.

Hertz joined the US Navy and left for active duty in April 1943. Means was floored by Hertz’s abrupt departure, but he agreed to allocate any new hyperthyroid patients to the study and follow-up their progress. Their conceptual understanding at that point was to attempt two rounds of oral RAI, for which Hertz deemed 12-15 mCi an appropriate activity. After which, patients with persistent symptoms were referred to a surgeon for possible thyroidectomy. Means was taken aback by Hertz’s unexpected departure and hired Earle Chapman to coordinate Hertz’s enrolled research patients at the MGH. Chapman, although he joined the group from his private clinical practice, actually attended the fateful lecture at the Vanderbilt Hall in December 1936 and followed the MGH-MIT progress with RAI through regular attendance at the weekly Thyroid Clinic meetings.

Hertz returned home to Brookline in the early 1945 and worked at the Chelsea Naval Hospital while attempting to re-enter the research circles at the MGH. Collegial relations had changed, and Hertz sensed it in the work atmosphere.Academic hierarchy, stained by personal ambitions and political sentiments, were blatant now. Hertz received a phone call in November 1945 from Morris Fishbein, the then editor-in-chief of *JAMA*, who confirmed Hertz’s ongoing suspicions. Fishbein in fact explained that the thyroid clinic had submitted a manuscript for a series of patients treated with RAI without consulting Hertz or including him as an author (16). *JAMA *initially rejected the paper for being excessively lengthy, and Hertz requested a copy in the interim while Chapman distilled the MGH manuscript down to an appropriate length. Understandably frustrated, Hertz wrote and submitted his own manuscript to *JAMA *with Arthur Roberts as a co-author. *JAMA* ultimately published both the articles, one by Hertz-Roberts and another by Chapman-Evans, side-by-side in the May 11, 1946 issue (17,18).^-^ Both the studies reached positive findings with RAI in the treatment of hyperthyroidism. Moreover, there was no patient overlap between the papers. In fact, the patients mentioned were in correspondence with Hertz during his active duty in the Navy, when Chapman was “really going to town”, were never included in Hertz’s series as promised. Chapman omitted to tell Hertz that he had slightly altered the experimental protocol, against the desire of Chief Means, to not administer a stable chaser dose of iodine after giving RAI, which added novelty to his series.

Despite the inner turmoil at the thyroid clinic, Hertz gained national recognition in other venues. He was inducted as a Young Turk to the American Society of Clinical Investigators (ASCI) in 1946 for his revolutionary work in developing the first theranostic modality in medicine: RAI treatment for thyroid disorders. ASCI inductees were called “young Turks” as an homage to the moniker for the members of the Committee of Union and Progress, a Turkish liberal reform movement in the Ottoman Empire that fought against Abdulhamid Han, the last Ottoman sultan to rule with absolute power. The sultan was deposed by the young Turks in the year 1909, when the ASCI held its inaugural meeting. The founding members based their new society on a revolutionary approach to research that emphasized newer physiological methods, with the hope of bringing sweeping reforms to research, which was analogous to the original young Turks in the Ottoman Empire (19).

## Early Days of RAI in Thyroid Cancer

Success with hyperthyroidism fueled a seamless, almost intuitive transition to the use of RAI in thyroid carcinoma. In the recent years, the Hertz family communicated that Saul Hertz planned to study RAI as a therapy for thyroid cancer treatment while at the MGH thyroid clinic (20,21).Indeed, Samuel Seidlin consulted Hertz in the year 1943 for his RAI expertise when managing a patient with metastatic thyroid cancer; this case later gained fame and was widely publicized on the national and international stages. Hertz articulated in 1946 that his research would focus on “cancer of the thyroid which I believe holds the key to the larger problem of cancer in general”, and that, after the War, “(new) demand expected in the fields of cancer and leukaemia for other radioactive medicines” (19).

The first research that focused on RAI in thyroid carcinoma were clinical reports led by a Berkeley physician-scientist Joseph G. Hamilton. A 1942 study by Hamilton’s team administered tracer radioiodine in 2 preoperative patients undergoing thyroidectomy (22). Pathological examination demonstrated diffused radioiodine avidity in the surgical specimen, although they found “no significant deposition” in malignant foci of the gland by autoradiography.

Metastatic thyroid carcinoma with avidity for radioiodine was first reported on April 3, 1942 by a group from the Columbia University (23). Although he was most interested in unrelated research on spirochetes, dermatologist Albert S. Keston was the lead author because he owned the Geiger-Müller counter they borrowed to study the RAI uptake. Their patient had known bone metastases and had the status of 35-years post-thyroidectomy. Keston et al. hand-scanned the patient together with the then radiotherapy resident Robert P. Ball on the morning of December 7, 1941; he later recalled that they learned of the attacks at Pearl Harbor mid-examination via radio broadcast (24). After locating a single functional metastasis in the right distal femur, they administered a therapeutic dose of 10-mCi RAI obtained from both the cyclotrons at Berkeley and at MIT. A 3-week follow-up tracer study demonstrated little uptake in the femoral lesion, hinting at a therapeutic role of RAI in functional metastases. However, when they later published an autopsy report for the same patient in 1944, they noted that the bulk of metastatic tumor burden proved to be undifferentiated tissue that “did not concentrate radioiodine” (25).

**Figure 5:**
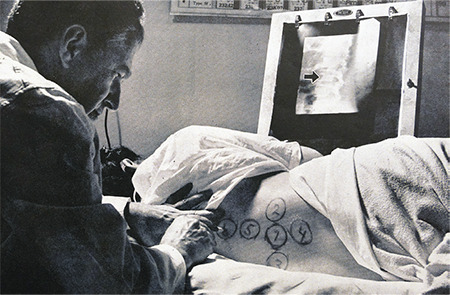
Samuel Seidlin examining a patient with a Geiger counter probe to detect RAI tracer uptake in metastatic thyroid lesions of the vertebrae in correlation with a radiograph of the spine (Life Magazine) RAI: Radioactive iodine

The aforementioned experiences seemed to dispel the possibility that RAI could “cure” thyroid cancer. However, the discussion was far from finished as the literature continued to evolve. On December 7, 1946, at the 5-year anniversary of the Pearl Harbor attack, *JAMA* published Samuel Seidlin’s extensive report of the first successful “treatment” of metastatic thyroid carcinoma with RAI (26). Seidlin (Figure 5) was an endocrinologist at Montefiore Hospital in New York when he consulted Hertz for an RAI advice in 1943. He subsequently followed his patient’s progress for 3 years before releasing the groundbreaking case. Seidlin’s coauthors in this case were Leonidas Marinelli, a physicist at Memorial Hospital (now Memorial Sloan-Kettering) who worked on dosimetry, and Eleanor Oshry, an applied physicist who was also the first woman to graduate from the Carnegie-Mellon’s engineering school.

Their landmark paper presented the extensive history of B.B., a then 51-year-old man with metastatic thyroid cancer. The patient underwent total thyroidectomy at the age of 30 years for a large substernal goiter that compressed his trachea. Microscopic examination of his surgical specimen revealed no normal structures, and the pathological diagnosis was “malignant adenoma”. Postoperatively, despite complete excision of the gland, B.B. did not become hypothyroid as was expected. In fact, he lived on for 15 years as a strong, healthy man before developing any classic symptoms of hyperthyroidism. His conditions of weight loss, palpitations, and anxiety progressively worsened until he was brought to surgical attention in November 1939 with a basal metabolic rate of +40 and a pulsatile tumor at the level of the twelfth thoracic vertebra. Accordingly, he underwent laminectomy at T12 and L1 for excisional biopsy, which revealed metastatic thyroid carcinoma. His postoperative course was complicated by thyroid storm, which in turn prompted his return for surgical neck exploration a few weeks later that turned out unremarkable.

B.B.’s condition worsened until his admission at the Montefiore Hospital on April 20, 1942, when he presented as a “small, emaciated, poorly developed man” standing at 4-feet, 10-inches tall and weighing 84 pounds. Seidlin’s clinical team treated B.B. with daily doses of 1-6-mL Lugol’s aqueous iodine for nearly 10 months to inhibit iodine organification through the Wolff-Chaikoff effect, thereby downregulating thyroid hormone production from his metastases. B.B. showed symptomatic relief at first, but, by January 1943, his condition worsened.

Seidlin consulted Saul Hertz soon after this incidence to discuss the possibility of using RAI to manage B.B.’s functional metastases. Hertz agreed with the management, and Seidlin only needed a contact for the supply of RAI. His decision to call Robey Evans at MIT was based solely on the fact that it was cheaper to call Massachusetts from New York than to connect with Berkeley, California. If that were not a factor, based on Hertz’s past professional disagreements with Evans, he would have probably recommended that Seidlin send his RAI inquiry elsewhere. Marshall Brucer, the past-president of the Society of Nuclear Medicine and cogent medical historian, later recalled an anecdote about Seidlin’s initial phone call with Evans, as follows:

Seidlin: “How much does it (RAI) cost?”

Evans: “Eighteen-hundred dollars an hour”.

Seidlin: (not expecting such a high price): “Send me some”.

Evans: “How many miliCuries do you want?”

Seidlin: “Send me a whole hour’s worth”.

When Brucer (27) wrote his 1966 *Vignettes in Nuclear Medicine*, he called this historic phone transaction “the beginning of radioisotope dosimetry”. The decision-making underlying their negotiation was driven by financial considerations more than scientific evidence. Seidlin later admitted that neither he nor his patient had $1.800 and that he did not understand what Evans meant by “miliCuries”. Eighteen-hundred dollars was possibly the operating price for the MIT cyclotron, and not the price for a therapeutic dose of RAI. Nevertheless, at this moment, Seidlin was a determined physician hoping to acquire a promising experimental therapy for his patient.

RAI shipments began arriving at the Montefiore Hospital from MIT, and Seidlin administered the first tracer dose to B.B. on March 11, 1943. Geiger-Müller counter hand-scanning confirmed all known metastases and revealed 2 new lesions: one focus was in the skull and another in the ischium. The first therapeutic RAI dose administered on May 11, 1943 contained 17 mCi of 12.6-h ^130^I with <0.5 mg of carrier iodine. Over the following 22 months, B.B. received a total of 16 therapeutic doses, of both ^130^I and ^131^I, the largest dose being 55.4 mCi, and the final dose was administered on March 6, 1945. The cumulative quantity of RAI administered throughout his clinical course totaled 268.8 mCi.

Urinary RAI excretion was tracked, and the specimens were often held for reclamation. Owing to the exorbitant cost of cyclotron-generated radionuclide, the Montefiore team extracted and recycled RAI from B.B.’s urine to re-administer by the oral route. On one such occasion, after administering the largest 55.4-mCi dose, they recovered 20.1-mCi from the urine and reused the rapidly decaying RAI. Although the laboratory assistants at Montefiore conducted the frugal practice of recycling RAI from urine for economic reasons, this practice later formed the basis for groundbreaking original research. One notable Montefiore laboratory assistant named Rosalyn Yalow translated the practice to albumin and insulin as well, and later won the 1977 Nobel Prize in Physiology or Medicine for her discovery of radioimmunoassay (28).

Seidlin’s (26) team noted marked clinical improvement in the early stages that steadily improved with successive treatments. B.B. gained weight and reached an equilibrium at 106 pounds by April 1944, occasionally commenting that he was “getting fat”. His bone pain drastically improved, he no longer had palpitations, and his basal metabolic rate dropped to -27. Seidlin’s clinic at the Montefiore Hospital followed B.B.’s case and were amazed by his subjectively well constitution, which seemed to represent that he was “cured” from metastatic thyroid carcinoma.

B.B. dropped his anonymity in 1949, coming out as Bernard Brunstein in the popular weekly *Life *Magazine. Seidlin organized this opportunity for the humble Brooklyn shoe salesman to share his story. The inspiring account portrayed Brunstein as having experienced near-death from metastatic cancer before he was recovered with the “atomic cocktail” regimen. The cover photo for the October 31, 1949 issue featured Princess Margaret, the younger sister of Queen Elizabeth II. The thyroid cancer article spanned 3 pages and included several photographs to accompany the 5 paragraphs of text. No authors were listed for the piece. The second-to-last sentence of this article mentioned that Seidlin had performed RAI treatment on 12 patients since 1942, 5 of whom “appear to be recovering”, presumably from their hyperthyroid state. Only 2 patients from that case series had tumors that appeared to stop growing.

RAI was brought to national attention and was presented as a cure for metastatic thyroid cancer based on the clinical improvement in just 1 patient. *Life *Magazine was arguably one of the most influential and widely-read weekly publication at the time. They covered all-important social topics ranging from World War II to U.S. presidential elections and served as a platform for famous authors, debuting important literature like Ernest Hemingway’s *Old Man and the Sea*. While Seidlin’s famous patient did improve clinically with respect to hyperthyroidism, *Life* somewhat misrepresented the claim that Seidlin “cured” metastatic thyroid carcinoma. In fact, only a little more than 2 years after this popular article, Brunstein reportedly died in 1952 from autopsy-proven anaplastic carcinoma (29).

Edward Siegel (30), the Physicist-in-Charge for Seidlin’s laboratory during 1949-1952, later wrote his recollection of the famous case. He described an interaction in which Seidlin became “frustrated and disappointed” when Brunstein initially failed to meet with the photographers from *Life*, saying to his patient, “Is this how you show your gratitude? After all, I cured you of cancer”. Brunstein replied, “Dr. Seidlin, you are supposed to be a smart man; if I had cancer five years ago, you know I’d be dead now!” (31). Essentially, even the patient himself believed that he was cured of something other than cancer, namely his hyperthyroidism.

Despite his interactions with the popular media, Seidlin himself was somewhat skeptical of having truly cured cancer. When examining the original December 7, 1946 *JAMA* case report, he wrote, “In spite of remarkable clinical improvement, it cannot be concluded that the functioning tumors have been completely destroyed, because recent tracer studies, although showing a marked increase in excretion, still show localization of RAI in these lesions” (26).

The *Harvard Crimson *ran a headline in May 1949 titled “Hertz to Use Nuclear Fission in Cure for Cancer”. On closer examination, the article discussed how “it has been demonstrated that the majority of cancerous thyroids do not take up the RAI” (32). Saul Hertz published a comprehensive chapter in January 1950, 2 months after the popular *Life* article. His commentary on RAI in thyroid carcinoma was captured best by the following statement: “On the whole the results of RAI treatment in cancer of the thyroid, while promising, have not indicated any great percentage of cures in the short time in which the procedure has been used” (33). He was well-aware of Seidlin’s patient and followed Brunstein’s treatment progress. Hertz recounted the case and added, “of course, it will take a number of years to demonstrate many such cases as this original one” (33).

## Building the Classic Age

Seidlin’s “radioablation” of thyroid cancer raised an important question about what is the appetite of thyroid tumors for RAI? A relevant autoradiography study appeared in the January 1950 issue of *Cancer* by a Sloan-Kettering group led by Fitzgerald et al. (34) Their paper discussed original research on the concentration of ^131^I in 100 consecutive cases of thyroid carcinoma, demonstrating that only 47 of the 100 patients had carcinoma with concentrated ^131^I (34). From the 100 patients, they studied 258 total lesions, and found that 47% had concentrated ^131^I. With respect to papillary thyroid carcinoma, the most common type, only 26% of the tumors demonstrated ^131^I uptake. The possibility that a tumor would concentrate radioiodine correlated with those follicular lesions that make functional colloid. This interesting research did not go unnoticed.

**Figure 6:**
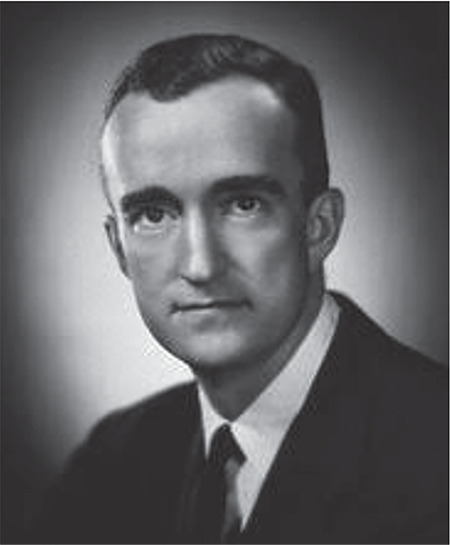
William Henry Beierwaltes, the pioneer nuclear medicine physician, 1955 (U.S. National Library of Medicine)

Beierwaltes et al. (35), one of the most pre-eminent nuclear medicine pioneer, cited the Fitzgerald paper in his authoritative 1957 book *Clinical Use of Radioisotopes*, stating that “less than 50 per cent of carcinomas of the thyroid have been demonstrated to pick up measurable amounts of RAI”. In several ways, Seidlin et al. (26) and Beierwaltes et al. (35) carried forward the foundation laid by Hertz with RAI in hyperthyroidism and built a conceptual basis for RAI in thyroid cancer. The rationale for applying RAI in thyroid cancer was clearly stated in 1957, in that “it is advisable to administer RAI to finish the job the surgeon started”. The purpose of RAI in thyroid cancer was to completely ablate the normal thyroid tissues to make surveillance easier. If any tumor remains *in situ* after surgical thyroidectomy that is amenable to RAI, it is an added benefit, but not the primary purpose. In fact, small clusters of thyroid remnant are a normal post-operative product of ligating vascular pedicles at the thyroid poles (36). Beierwaltes (35) (Figure 6) reflected on the role of RAI in thyroid cancer in his autobiography *Love of Life*: “We were the first to insist on a total surgical thyroidectomy as the first step in treating all patients with thyroid cancer. The second step was to keep the patient off thyroid hormone for six weeks. The third step was to do a radio-iodine scan of the neck and whole body to look for thyroid remnant or metastasis. Then we gradually found that the usual effective dose of radio-iodine treatment was (1) 150 mCi to ablate the thyroid remnant; (2) 175 mCi for regional node metastasis; (3) 200 mCi for spreads outside the neck” (37). Beierwaltes provided the original framework, but the methodology for RAI ablation in cancer became morphed in subsequent decades and continues to remain debatable until date.

## Leaning into the Age of Genomics

Molecular theranostics is the new paradigm in thyroid cancer risk stratification. More refined classification schemes based on genomics and their downstream phenotypic expressions are currently being formulated. Genomics with molecular pathology and molecular imaging reflect the true biological nature of different cancer types that are currently defined by the conventional morphologic features. The tumor differentiation/de-differentiation and clinical behavior for each individual cancer are now definable by molecular markers in addition to the standard morpho-pathology. In 2014, the first comprehensive study on genomic characterization of thyroid cancer was published, which involved compiling of large data on the morphological and molecular features of papillary thyroid cancer (38). This work marked the beginning of a new paradigm for thyroid cancer diagnosis and management. First and foremost, this study identified pathways of thyroid cancer oncophysiology and their impact on iodine metabolism. Correlations between morphology and driver genetic mutations as well as thyroid differentiation score were first clearly described in a systematic fashion with this study. It is clearly evident that the conventional postoperative risk stratification criteria were to vacate their roles in molecular diagnostics and in the state-of-the-art RAI theranostics. This new paradigm promises to resolve the long-winded equipoise and facilitate research in optimal care of thyroid cancer patients.

## Epilogue

Nuclear Oncology has an inspiring and thought-provoking history. This article is a tale of its “Ontology”, the core philosophy behind the concept of theranostics. Thyroidology has evolved into a new paradigm with advances in genomics and molecular medicine. The molecular theranostic profiles are expected to be incorporated into dynamic clinical decision-making and management algorithms for thyroid surgery and RAI therapy remaining faithful to the ontology of nuclear oncology.
